# Association of environmental traits with the geographic ranges of ticks (Acari: Ixodidae) of medical and veterinary importance in the western Palearctic. A digital data set

**DOI:** 10.1007/s10493-012-9600-7

**Published:** 2012-07-28

**Authors:** A. Estrada-Peña, Robert Farkas, Thomas G. T. Jaenson, Frank Koenen, Maxime Madder, Ilaria Pascucci, Mo Salman, Jordi Tarrés-Call, Frans Jongejan

**Affiliations:** 1 Department of Parasitology, Faculty of Veterinary Medicine, Miguel Servet, 177, 50013 Zaragoza, Spain; 2 Department of Parasitology and Zoology, Faculty of Veterinary Science, Szent István University, 1078 Budapest, Hungary; 3 Medical Entomology Unit, Department of Systematic Biology, Evolutionary Biology Centre, Uppsala University, Norbyvägen 18d, 752 36 Uppsala, Sweden; 4 Interaction and Surveillance Directorate, CODA-CERVA (Veterinary and Agrochemical Research Centre), Groeselenberg 99, 1180 Ukkel, Belgium; 5 Institute of Tropical Medicine, Antwerp, Belgium; 6 Department of Veterinary Tropical Diseases, Faculty of Veterinary Science, University of Pretoria, Private Bag X04, Onderstepoort, 0110 South Africa; 7 Istituto Zooprofilattico Sperimentale dell’Abruzzo e del Molise “G.Caporale”, Campo Boario, 64100 Teramo, Italy; 8 Animal Population Health Institute, College of Veterinary Medicine and Biomedical Sciences, Colorado State University, Fort Collins, CO 80523-1644 USA; 9 European Food Safety Administration (EFSA), Parma, Italy; 10 Utrecht Centre for Tick-borne Diseases (UCTD), Faculty of Veterinary Medicine, Utrecht University, Yalelaan 1, 3584 CL Utrecht, The Netherlands

**Keywords:** Ixodidae, Distribution, Western Palearctic, Compilation

## Abstract

We compiled information on the distribution of ticks in the western Palearctic (11°W, 45°E; 29°N, 71°N), published during 1970–2010. The literature search was filtered by the tick’s species name and an unambiguous reference to the point of capture. Records from some curated collections were included. We focused on tick species of importance to human and animal health, in particular: *Ixodes ricinus*, *Dermacentor marginatus*, *D. reticulatus*, *Haemaphysalis punctata*, *H. sulcata*, *Hyalomma marginatum*, *Hy. lusitanicum*, *Rhipicephalus annulatus*, *R. bursa*, and the *R. sanguineus* group. A few records of other species (*I. canisuga*, *I. hexagonus*, *Hy. impeltatum*, *Hy. anatolicum*, *Hy. excavatum*, *Hy. scupense*) were also included. A total of 10,280 records was included in the data set. Almost 42 % of published references are not adequately referenced (and not included in the data set), host is reported for only 61 % of records and a reference to time of collection is missed for 84 % of published records. *Ixodes ricinus* accounted for 44.3 % of total records, with *H. marginatum* and *D. marginatus* accounting for 7.1 and 8.1 % of records, respectively. The lack of homogeneity of the references and potential pitfalls in the compilation were addressed to create a digital data set of the records of the ticks. We attached to every record a coherent set of quantitative descriptors for the site of reporting, namely gridded interpolated monthly climate and remotely sensed data on vegetation (NDVI). We also attached categorical descriptors of the habitat: a standard classification of land biomes and an ad hoc classification of the target territory from remotely sensed temperature and NDVI data. A descriptive analysis of the data revealed that a principal components reduction of the environmental (temperature and NDVI) variables described the distribution of the species in the target territory. However, categorical descriptors of the habitat were less effective. We stressed the importance of building reliable collections of ticks with specific references as to collection point, host and date of capture. The data set is freely downloadable.

**Electronic supplementary material:**

The online version of this article (doi:10.1007/s10493-012-9600-7) contains supplementary material, which is available to authorized users.

## Introduction

The world’s major tick collections house hundreds of thousands of specimens. Each specimen bears at least in theory one label on which the metadata for that specimen are recorded. These data collectively document much of what is known about the diversity, geographic distribution, and phenology of ticks around the world. Thus, they have the potential to serve as baseline from which to document, among other things, the natural occurrence of ticks as currently reported. Although these data are nominally accessible to researchers, for all practical purposes they are unavailable to the general community. As a result, the investments that have been made in acquiring, processing, and storing the specimens and their data often provide little in scientific knowledge. In the publication process, the link to the underlying primary data is usually broken. After publication, data from newly collected material (frequently inspired by a piece of research) often cannot be incorporated into the collective understanding. A new suite of tools, based on biodiversity informatics, as well as resources summarizing environmental and climate information are being increasingly used to address the relationships of living beings with their surrounding environment (Flemons et al. 2007; Magurran 2004). These tools can effectively be applied to reduce the limitations of tick collections and reports, and make available the information for researchers.

In order to be useful in public health and veterinary medicine, the study of the geographical distribution of ticks requires centralization of published reports. The only thoughtful efforts to compile tick distribution were made through the former web site of the European Union-funded and concerted action, International Consortium of Ticks and Tick-Borne Diseases (ICTTD), that hosted tabular data for more than 30,000 records of ticks in the world, and the compilation by Cumming (1999) which focused on the Afrotropical region. There is a need to understand which physical and biological key factors determine the geographical ranges of different tick species. It is also essential to expertly curate the compiled records to avoid misidentification or compilation of records that are incorrectly reported. The increasing evidence surrounding the importance of ticks as vectors of pathogens affecting human and animal health has led to a rise in the frequency of publications devoted to reporting findings about tick distribution, tick phenology, tick hosts and pathogens that ticks can harbor (EFSA 2010). When an area has been surveyed for ticks, geographical plotting and correlation analysis with certain environmental parameters permit certain inferences to be drawn concerning what ticks may be present, where and when they may occur, and possibly even why they are present in a particular area. When detailed information is available on the main ecological preferences of a species, inferences may be made as to the presence or absence of the species in non-surveyed areas.

This project began with the purpose of collecting, mapping and making available to researchers the recorded geographical distribution of the ticks that are of medical and veterinary importance in the different regions of the world. The project started under the umbrella of two concerted actions of the European Union. Under the auspices of the European Food Safety Administration (EFSA) the data were updated by analyzing reports on ticks in the western Palearctic, published in the years 2000–2010. This paper is based on these tick records, which are complemented by a set of quantitative and categorical variables aimed to describe the habitat at every collection site. The aim of this paper is to build a digital data set of tick records in the Western Palearctic, and to give an overview of the environmental characteristics in the geographic range of each one of the more abundantly represented tick species. The data set is downloadable from several sites.

## Materials and methods

### Assembly of tick records

Western Palearctic is here considered to include a region outlined between 11°W to 45°E and 29°N to 71°N. From an administrative point of view, it incorporates every country in the European Union, and to the north of the Sahara desert, to the Atlantic Ocean. The Canary Islands were included in the surveyed territory but Iceland and The Azores were not. No ticks have been reported from Iceland and only a few records exist for Azores, without adequate reference to a pair of coordinates.

A first effort to compile the bibliographical information was done by a systematic search of the published, peer-reviewed literature using online scientific bibliographic data bases covering the period since, approximately, the year 1970. Such a first step was finished in 2004 and resulted in a compilation of the available records in gridded maps at low resolution (Estrada-Peña et al. 2004). This compilation was completed by a systematic analysis of the peer-reviewed literature published between 2000 and 2010 (EFSA 2010). The basic criteria included in both searches were the taxonomic genus or family names of the ticks as found in the title or the abstract of the paper. Further reading of each reference searched for information about the species name of the tick and a strict reference to a site of capture. The site may have been referred to by its coordinates or to which name provided enough information to locate it in digital gazetteers or regional maps. Records were not included if they referred only to a genus name or reported from a large administrative division (e.g. a province), or if the locality information was ambiguous (i.e. if several localities in the same country had this same name, without further reference to a province). Literature searches were concluded on 31 October 2010 and all citations meeting our search criteria were reviewed. If available, additional information gleaned from the published reports included the scientific name of the host and the date of capture.

Unpublished records of ticks available in some curated collections were also considered for inclusion. For logistic reasons, it was not possible to study all major museum collections of ticks in the target territory. However, since we wanted to include as much information as possible, we included records from the collections which were readily accessible. The tick names in the final data set conform to those provided in Guglielmone et al. (2010). Every record was subjected to the opinion of a panel of experts (see EFSA 2010). Records considered as potentially incorrect, based on the current knowledge of the distribution of ticks in the Palearctic region, were removed from the data set.

We focused on the following tick species of importance for human and animal health: *Ixodes ricinus*, *Dermacentor marginatus*, *D. reticulatus*, *Haemaphysalis punctata*, *H. sulcata*, *Hyalomma marginatum*, *Hy. lusitanicum*, *Rhipicephalus annulatus*, *R. bursa*, and the *R. sanguineus* group. The lack of reliable species identification of many specimens in the sanguineus group, and the inherent difficulty in separating two species of the group (*R. turanicus* and *R. sanguineus*), have loaded the scientific literature with a wealth of unreliable information. Therefore, these records were classified as belonging to the sanguineus species complex. Other species reported in the region were included in the data set of tick occurrences, but they constituted only a small fraction of the total number of records. These species are *I. canisuga*, *I. hexagonus*, *Hy. impeltatum*, *Hy. anatolicum*, *Hy. excavatum*, *Hy. scupense* (including the former *Hy. detritum*, now considered a synonym of the later, Guglielmone et al. 2010) and *I. gibbosus*. The aim of including these species here is to provide as many reliable records as possible for future reference. However, the scarcity of such records prevented any further analysis. The complete data set is downloadable from the journal’s site as Supplementary material.

### Descriptors of tick occurrence

The geographic range of a tick is rarely correlated with administrative divisions. The distribution of an organism should be related to environmental (abiotic and biotic) factors. However, there is a lack of harmonization of descriptors of tick occurrence. Because the increasing interest in species distribution models for ticks, we wanted to provide with both a consistent set of tick records and the potential descriptors of tick occurrence, aimed at prospective mapping. We wanted to use remotely sensed environmental features together with both interpolated gridded data and categorical descriptors of the territory. Each record was loaded with monthly values of temperature and rainfall obtained from the interpolated grid at www.worldclim.org (Hijmans et al. 2005; accessed September, 2011) at a resolution of 5 km. These monthly values included minimum and maximum mean monthly temperatures as well as mean monthly rainfall. Such climate information has been prepared with means for the period 1960–1990. We also added monthly information on Land Surface Temperature (LST) and the Normalized Derived Vegetation Index (NDVI) at a resolution of 5.6 km. as recorded by the MODIS series of satellites. NDVI is an indicator of the vegetal stress and is therefore an accurate measure of vegetal phenology adequate to describe tick occurrence (Estrada-Peña 1999). The ten-year (2000–2010) monthly series of LST and NDVI was downloaded from http://modis.gsfc.nasa.gov/ and monthly averaged values were attached to each tick record. Both LST and NDVI are provided to complement gridded climate values. However, they are available only since the year 2000, while some records of ticks were reported as early as 1980.

Tick occurrence by a categorical description of the habitat has already been described (e.g. Gilot et al. 1994, 1996). The following two classifications were provided with our data set. One was obtained from http://www.worldwildlife.org/science/data/item1875.html (accessed September, 2010), and it shows the major land biomes of the earth. Its purpose is to describe the main regions of the earth, not tick habitat. The second classification originates from satellite-derived data on LST and NDVI. We used data for 2000–2010 to produce an unsupervised classification of the target territory by a k-means algorithm in the software package (Erdas Imagine 9.1). The classification was intended to produce a set of spatial categories based on the seasonal pattern of both LST and NDVI. Each resulting category has unique seasonal patterns of LST and NDVI and this is of interest to categorize the geographic distribution of ticks. Each record was thus labeled with information about the category of the habitat. Figure S1 of the supplementary material shows the geographical range of each category derived from climate data. Table S1 contains statistical information about the values of LST and NDVI as recorded for each category.

### Additional analyses

In order to analyze the geographic occurrence of ticks and associated environmental descriptors, we tabulated the number of records for each tick species followed by a cross tabulation between the species of ticks and the hosts reported in the literature review. We then aimed to describe the recorded distribution of ticks under a quantitative and a categorical set of environmental descriptors. We derived this descriptive analysis from remotely sensed information (according to Hay et al. 2006), which is also attached to the distributed data set of records. The *fundamental niche* of a species includes the total range of environmental conditions suitable for existence. The fundamental niche thus excludes any influence of interspecific competition, predation, dispersal limitation, and natural or human disturbances (Pulliam 2000). The *realized niche* is that part of the fundamental niche which is actually occupied by the species under these constraints. It defines the spatial distribution of a species in a community and in a study area (Guisan et al. 2006). The realized niche of each species is thus confined by the maximum and minimum values of the reduced climate found across the entire study region.

There has long been a tendency to consider a Principal Components Analysis (PCA) as a consistent description of a climate niche (i.e. Thuiller 2004; Araújo et al. 2005; Broennimann et al. 2007). This has already been applied to ticks (i.e. Estrada-Peña and Venzal 2007; Estrada-Peña 2008), although other authors prefer to synthesize information by a Fourier analysis of these environmental variables because seasonal information is lost in classic PCA (i.e. Rogers et al. 1996, 2002; Randolph et al. 2000). In both cases the number of original variables is reduced while maximum variability of the original data is retained. We considered the first three axes of a PCA of the monthly averaged LST and NDVI values as definition of the climate space of the ticks. In principle, climate space occupancy by ticks could be analyzed with a greater dimensionality, but in practice increased dimensionality brings greater challenges for interpretation. These first three axes explained 82.4 % of the variability. Axis 1 was loaded by, and was inversely related to, the average annual LST. Axis 2 was inversely correlated with the range between maximum and minimum LST; large seasonal amplitude in temperature is related to negative values in this axis. Axis 3 was inversely related to mean NDVI. We plotted the occurrence of ticks under such a reduced space, looking for explicit associations between species and environmental traits.

We further examined the amount of niche overlap among tick species, using the set of PCA-derived “coordinates” of each species in the dimensions of the climate space. These “coordinates” were obtained from the PCA values at each occurrence point. Niche overlap between any two species involves three steps: (1) calculation of the density of occurrences and climate factors along the axes of the multivariate analysis before (2) measurement of niche overlap along the gradients of this multivariate analysis and (3) statistical tests of niche equivalency. The climate space was divided into a grid of × r cells of a three-dimensional volume, each cell being a unique vector of climate conditions present at one or more sites of the geographic space. The occurrences of each species in each unique cell of the grid were used to map the occurrence of the ticks in the climate space. The number of occurrences of a tick species is dependent on sampling strategy, and a compiled data set of tick distribution may not entirely reflect the actual distribution of species. We adhered to published methods (Broennimann et al. 2012) and applied a standard Gaussian kernel to determine the smoothed density of occurrences along the environmental axes. We built from the methodology previously described (Warren et al. 2008) to perform niche equivalency tests using the D metric (Schoener 1970). The niche equivalency test determines whether niches of two entities in two geographical ranges are equivalent (i.e. whether the niche overlap is constant when randomly reallocating the occurrences of both entities among the two ranges). Full details of the procedures are available in Warren et al. (2008).

## Results

Globally, the literature search resulted in 2,566 references containing potential data to be reviewed. Of these publications, 1,116 fulfilled the inclusion criteria, providing at least the binomial name of the species and the coordinates of the record or an unambiguous name of the collection site. A total of 10,280 records of ticks (from published sources and curated collections) were regarded as appropriate to be included in the data set. Table 1 shows the number of records of each species of tick in the final data set. An additional 7,412 records of ticks were not included because they were referred only to large administrative divisions (provinces, countries) and were thus not possible to associate with a particular collection site. *I. ricinus* (44.3 %) was the predominant species in the data set; *H. marginatum* and *D. marginatus* accounted for 7.1 and 8.1 % of the records, respectively. The *R. sanguineus* group accounted for about 4 % of the total records.

**Table 1:** Total number of records for each tick species as recorded in the western Palearctic, after a literature survey updated with data from curated collections and covering approximately the years 1975–2010

Species	N	%
*Dermacentor marginatus*	734	7.1
*Dermacentor reticulatus*	882	8.6
*Haemaphysalis punctata*	479	4.7
*Haemaphysalis sulcata*	298	2.9
*Hyalomma anatolicum*	12	0.1
*Hyalomma excavatum*	211	2.1
*Hyalomma impeltatum*	8	0.1
*Hyalomma lusitanicum*	526	5.1
*Hyalomma marginatum*	787	7.7
*Hyalomma scupense*	135	1.3
*Ixodes canisuga*	8	0.1
*Ixodes hexagonus*	7	0.1
*Ixodes ricinus*	4,554	44.3
*Rhipicephalus annulatus*	201	2.0
*Rhipicephalus bursa*	960	9.3
*Rhipicephalus sanguineus *group	478	4.6
Total	10,280	

A high percentage (60 %) of the records of *Hy. excavatum* were unreliably georeferenced. As a result, they were used only for assessment of host usage

**Fig. 1:**
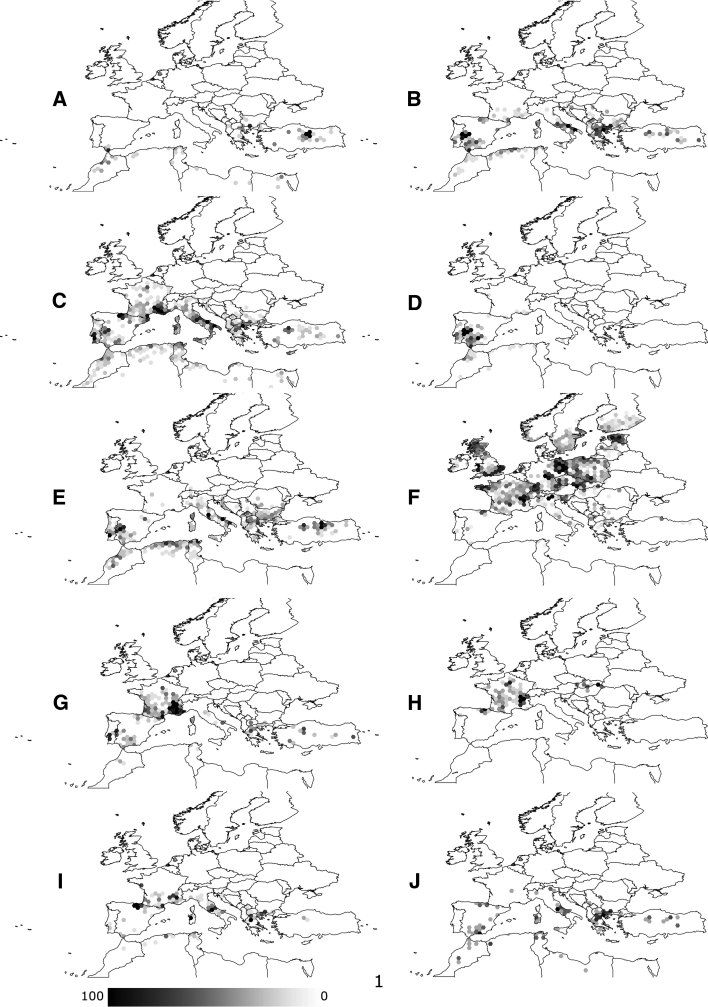
The distribution of tick species in the geographical space of the western Palearctic, plotted as the percent of records for every hexagonal cell of 0.5° of spatial resolution. **a** *R. annulatus*, **b** *R. bursa*, **c** *R. sanguineus*, **d** *Hy. lusitanicum*, **e** *Hy. marginatum*, **f** *I. ricinus*, **g** *D. marginatus*, **h** *D. reticulatus*, **i** *H. punctata*, **j** *H. sulcata*

The literature survey reported the occurrence of immature stages most often for *I. ricinus* (78 % of the examined references reporting such species). For the other tick species, the most often reported stage (collected by dragging and from hosts) was the adult. Overall, 96 % of the valid references for non-*I. ricinus* species reported only adults. Only 18 % of the surveyed literature contained information about the procedures used for identification of tick species. Thus, in 82 % of the references the reader is left unaware of the taxonomic criteria used to diagnose the tick species. This is likely to generate concern about the identity of confusing species, mainly for the immatures instars. In the case of *I. ricinus*, only 3.9 % of available references explicitly stated the efforts to separate the species from other ticks occurring on the same hosts. Figure 1 summarizes the recorded occurrence in the Western Palearctic of the ten tick species for which enough records were compiled. Records are plotted as percentage of records of the species in individual hexagonal cells of 0.5° of spatial resolution. The maps produced should not be used for the tracking of every single tick record. Such a basic plot outlines three groups of species according to their occurrence ranges: those restricted to the Mediterranean area (*R. bursa, R. annulatus, Hy. marginatum, Hy. lusitanicum, H. punctata, H. sulcata*); one species occurring over extensive areas of the Palearctic (*I. ricinus*); and another one mainly inhabiting regions with a mild Atlantic climate (*D. marginatus*). It should be noted that both *R. sanguineus* and *R. turanicus* were grouped together in the same map.

Information on hosts was available for 6,325 records (61 %). No efforts were made to check the reliability of the determination of the hosts and we only summarized such information following the host taxonomic Order (Table 2). It revealed the high reporting of every species on domestic ungulates (Artiodactyla), with the exception of the species in the *R. sanguineus* group and *I. ricinus*, which were commonly reported on Carnivora. Interestingly, more than 50 % of records of *I. ricinus* were collected by flagging/dragging, usually during parallel studies on carried pathogens. Species most often reported on humans were *D. marginatus* and *I. ricinus*. The information in Table 2 should be considered as summarized information and not an evaluation of host preferences.

**Table 2:** An overview of the patterns of occurrence of ticks in the western Palearctic according to the hosts’ systematic order

Species	Artiodactyla (Bovidae)	Artiodactyla (Ovidae)	Artiodactyla (Suidae)	Artiodactyla (Camelidae)	Perissodactyla	Carnivora	Rodentia	Insectivora	Lagomorpha	Hominidae	Aves	Reptilia
*D. marginatus*	20.45	39.67	16.36		6.34	2.25	4.70	0.61	0.41	9.20		
*D. reticulatus*	11.11	11.46	2.08		10.42	58.68			0.00	6.25		
*H. punctata*	24.34	44.39	0.48		4.06	3.34	0.24	0.48	1.19	0.24	21.24	
*H. sulcata*	10.42	65.63	3.13		3.13	4.69				0.52	3.65	8.85
*Hy. excavatum*	65.70	11.11	1.45	9.18	3.38	0.48	6.76	1.93				
*Hy. lusitanicum*	28.14	51.26	13.57	0.25	0.75	1.01		0.25	2.01	2.26	0.50	
*Hy. marginatum*	61.54	7.28	3.02	0.82	13.87	0.27	0.14	0.27	1.92	0.14	10.71	
*Hy. scupense*	86.67	11.11								2.22		
*I. ricinus*	20.51	22.31	2.44		1.67	28.08	5.26	2.05	3.85	7.95	4.36	1.54
*R. annulatus*	90.59	6.93			0.99	0.99			0.50			
*R. bursa*	38.12	47.23	2.57		7.23	3.27	0.30	0.10	0.79	0.30	0.10	
*R. sanguineus*	5.72	9.59	0.37		0.18	74.17	3.32	3.14	1.48	1.85	0.18	
*R. sanguineus *group	10.2	13.9	14		12.4	37.1	24.1	35.4	25	9.55	2.26	
*R. turanicus*	26.63	30.10	5.27		6.38	22.47	2.64	2.22	2.77	0.97	0.55	
Totals	30.2	28.0	4.0	0.3	5.0	21.7	2.1	1.3	1.7	2.4	2.9	0.4

Data represent the percentage of the records of each species (rows) as reported on different taxa of hosts. The last row (Totals) indicates the percentage of total records of ticks on each host order

**Fig. 2:**
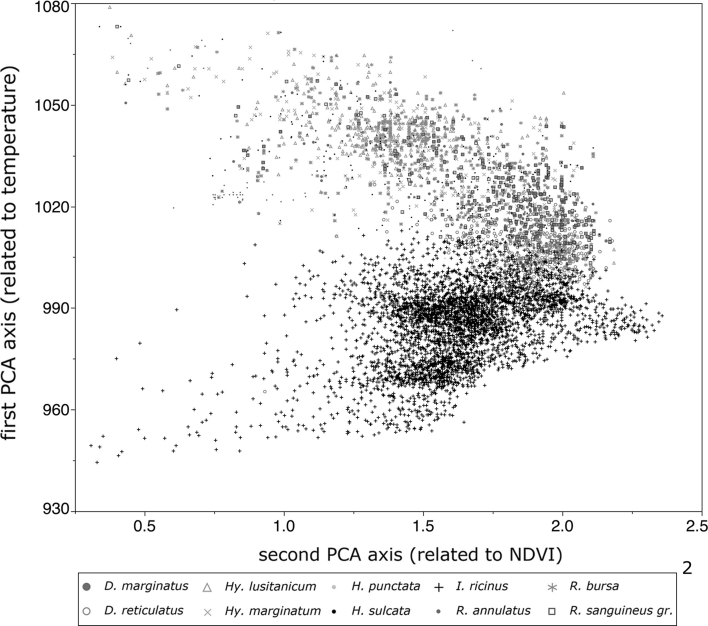
The distribution of tick species in the climate space of the western Palearctic, plotted as the position of every record in a reduced climate space. Climate (temperature and Normalized Derived Vegetation Index [NDVI]) values were reduced using a principal components analysis and original variability was reduced to 3 axes. Only the first and second axes are illustrated. They are related respectively to mean annual temperature and mean annual NDVI. Each tick record is a single point on the figure

Figure 2 plots the occurrence of the ticks along the two first axes of the reduced climate space resulting from PCA (rather than in the geographical one). Thus, records are plotted against the first (directly related to temperature) and second (directly correlated with NDVI) axes of a PCA of the monthly values of temperature and NDVI. Such an analysis allows for a strict comparison of the gradients separating the occurrence areas of the ticks in Western Palearctic as well as the distributional amplitudes of the species on these main gradients. It supports the main results of geographical occurrence outlined before, and provides a coherent background against which to test the climate data as recorded at the collection sites. The primary European-Mediterranean division is observed in the plot as a clear border between the recorded occurrence of *I. ricinus* (bottom) and the occurrence of the rest of species (top). In this sense, both *D. reticulatus* and *D. marginatus* occur in an intermediate zone between the other strictly Mediterranean species on one hand and *I. ricinus* on the other. Both groups of species occur together along a relatively narrow range of temperature-derived values and a large range of values of NDVI. It is interesting to notice that the range of temperatures under which *I. ricinus* has been reported is as large as the total range of temperature for the occurrences of the other species. In other words, *I. ricinus* has been reported in a temperature envelope as large as the sum of the envelopes of all the other species combined. Table 3 displays the niche similarity values computed from the set of occurrences, using the metric D. This analysis measured how the climate niches of every two species of ticks overlap in HIT the reduced PCA values. In other words, the analysis shows how similar is the climate niche in which the ticks have been reported. There is a relatively high but variable amount of niche overlap for Mediterranean species. However, *I. ricinus* has the lowest values of niche overlap with every other species. These values support previous results on the large split of species occurring along Mediterranean and European habitats. They also underpin the validity of a dimensionally reduced climate data set to quantitatively define the occurrences of the ticks and provide a comparative background. It is noticeable the high niche overlap verified between *H. punctata* and the *R. sanguineus* group of species.

**Table 3:** The Schoener’s D index for the niche overlap of the target tick species

	*Hy. marginatum*	*Hy. lusitanicum*	*H. punctata*	*H. sulcata*	*R. annulatus*	*R. bursa*	*R. sanguineus*	*I. ricinus*
*D. marginatus*	0.206	0.196	0.479	0.612	0.16	0.29	0.479	0.244
*Hy. marginatum*		0.571	0.576	0.617	0.615	0.791	0.574	0.050
*Hy. lusitanicum*			0.453	0.201	0.444	0.588	0.455	0.067
*H. punctata*				0.355	0.432	0.649	0.971	0.126
*H. sulcata*					0.415	0.312	0.412	0.054
*R. annulatus*						0.515	0.43	0.040
*R. bursa*							0.647	0.073
*R. sanguineus*								0.125

A higher (in the range 0–1) index indicates a higher climate niche overlap for the pair of species. We removed *D. reticulatus* from this analysis because it was assumed that the distribution of the species was fully captured by the analysis of the literature

We also aimed to report the recorded distribution of ticks, as described by categorical classifications of the habitat. Supplementary Table S2 summarizes the occurrence of ticks along a standard classification of the landscape in land biomes. The tabulations reflect a relatively restricted occurrence of a few species, like *D. reticulatus*, *Hy. lusitanicum* and *R. annulatus* to some ecological regions (Western European Broadleaf Forests, Iberian Sclerophyllous and Semi-Deciduous Forests and Central Anatolian Steppe And Woodlands, respectively). The rest of the species are loosely related to the biomes in the western Palearctic. A coherent picture of the associations of the ticks with specific land biomes cannot be obtained at the resolution of the classification, which is the highest available for a wide geographical range. Supplementary Table S3 summarizes a similar view when cross-tabulating tick records against a classification of the habitat from remotely sensed features of both temperature and NDVI (see Supplementary Figure S1 for information about the geographical range of each category). While the different tick species seem to be more coherently attached to single features of the territory, this is because of the lower number of categories.

## Discussion

We presented the information about the reported distribution of ticks in the western Palearctic since approximately the year 1970, collated mainly as a bibliographical search. The generated data set also included information from some curated collections in institutions, totaling more than 10,000 records with reliable geographical information. Areas where ticks have not been reported does not necessarily reflect their absence. A considerable amount of data on tick distribution has been accumulated over the years, but much of this information was obtained incidentally from studies undertaken for other purposes and with other objectives. Therefore, it is for the most part ecologically incomplete. Along with the absence of much ecological information, many of the available records are brief and/or incomplete in other ways. For example, it is not uncommon to report the site of collection as a broad geographical or political area, without the possibility to link reports and the environmental features. Others, though they may indicate a more precise locality, do not mention the host. Some published records list a series of localities followed by a list of hosts, making it impossible to relate any specific host within this list to the specific locality wherein the tick was collected. Data as to the time of collection of ticks are often incomplete and make the information virtually useless in attempting to assess the phenology of the tick. Thus, much of the available information on the distribution of ticks allows not much more than a mere geographical plotting and serves only to indicate the approximate occurrence of certain species. As such, this information has limited value.

Our compilation ran along the efforts by Pietzsch et al. (2005) to assimilate published records of *I. ricinus* in the United Kingdom, but was applied to a wider ecological unit such as the western Palearctic. Several problems of homogeneity were detected in the available references and records. First, many published reports and/or curated collections did not specifically record the collecting locality or the coordinates. This is a particularly serious problem regarding the tick *D. reticulatus*. Visual examination of the reliable records of that species shows a gap in wide regions of central Europe where the tick has been previously reported (Dautel et al. 2006; Nijhof et al. 2008; Srétér et al. 2005; Bullová et al. 2009), or for which adequate records have been produced after the conclusion date of our literature survey. Another potential drawback is the collection pressure affecting the reported prevalence. Ticks have been collected along the interests of research groups involved in the study of the ticks and tick-transmitted pathogens. This produced an obvious bias towards particular areas and species. The recognized importance of *I. ricinus* and its role in the transmission of pathogens (i.e. EFSA 2010) are clearly perceived in the data set, since the species represents more than 48 % of the total records. This affects the homogeneity in the compiled data set regarding the species composition and the local or regional importance of the ticks.

The compilation lacks a time label for most references. Recent reports included a mention of the year of capture, but such an allusion is missing in as many as 8,669 records. Absolute date of collection (calendar day) was available for only 651 records. Therefore, it has been impossible to prepare a homogeneous temporal background against which to test the concerns on the geographical spread of some species (i.e. Danielova et al. 2006; Jaenson et al. 2012). Together with already published reviews on tick distribution (i.e. Jaenson et al. 1994; Jore et al. 2011; Siuda 1993) we hope to produce a framework against which future records of ticks could be examined in relation to climate and other environmental traits. We thus aimed to provide environmental information in the neighborhood of each record. We gathered such information from remotely sensed sets of temperature and vegetation, reflecting the prevailing views about their importance in outlining the environment as perceived by the tick. As stated by Broennimann et al. (2012) a rigorous comparison of climate niches among species can be done only in the environmental space as opposed to other interpretations made under the geographical aspect, thereby correcting for bias associated with geographical dimensions. We already provided a geographical background (Estrada-Peña et al. 2004; EFSA 2010), and this data set supplied quantitative and categorical features to test the occurrence of ticks. The information included in the compiled data set is intended to be as much complete as possible, covering a relatively long period. It is however necessary to stress the possible lack of temporal coherence between the records of ticks and the gridded and satellite-derived variables issued with them. While the gridded variables are the mean of a relatively long lasting series (1960–1990), the MODIS data came from the period 2000–2010. This data set is intended to be a summarizing point against which delineate prospective collections in the target territory. How the climate niches of ticks change across space and time is fundamental to our understanding of many issues in the epidemiology of ticks and tick-transmitted pathogens. Such knowledge has practical importance as researchers are increasingly asked to forecast invasions or changes in species distributions under climatic change (i.e. Cumming and Van Vuuren 2006).

Classification of the habitat along standard land biomes might not provide the strict background necessary to discriminate the factors driving tick distribution, which are quantitative and variable in nature (i.e. Estrada-Peña and Venzal 2007). Classifications based on land biomes tend to be similar across a large geographical range even if driven by similar climate traits (Hazeu et al. 2011). While these classifications may be of utility at a local or regional scale, it is difficult to extrapolate comparative patterns when they are applied to wide regions. This is why contemporary attempts to build categorical classifications of the habitat have focused on the use of quantitative climate traits, following a protocol of reduction of redundant data (Metzger et al. 2005). Our descriptive results support the use of quantitative data sets instead of categorical ones as more rewarding to better capture these drivers. The distribution of ticks, however, changes over time perhaps in a cyclical fashion, or perhaps in response to more general, long-term factors induced by human population growth or climate change. Tick distribution, therefore, may not be adequately expressed as a static area, but as a probability surface, and those probabilities may fluctuate through time. We thus implemented a categorical representation of the environmental traits over the Western Palearctic based on quantitative, remotely sensed features, with the accepted scientific understanding that climatic factors are the main determinants of ecosystem patterns on a continental scale (Klijn and De Haes 1994). Previous approaches (i.e. Mücher et al. 2010) did not consider Northern Africa; this is of importance because the tick species in western Palearctic form a structural assemblage (Morel 1965) requiring an integrated study. Such classification allows for greater levels of recursion than those presented here and can be applied to narrower time windows, allowing the capture of trends for these traits and consequently the shift in drivers of importance for tick occurrence. It remains of interest to examine its potential use in smaller regions for which adequate and homogeneous sets of tick records exist.

This paper introduced the first digitally-available collection of tick records in the western Palearctic, together with harmonized information of environmental traits. It is available at http://sites.google.com/site/palticks. A need exists for detailed and reliable information on the distribution for all species of ticks, not only because of the interest in human and animal health, but also for purely biological considerations. We strongly encourage prospective authors to prepare and deliver complete lists of records, and not only printed maps, to allow such monumental works to be adequately included into data sets over large geographic ranges. It is also necessary to call attention to the need for accurate identification of the ticks collected in order to reduce confusion in this field of research. The report of taxonomic names not accepted by the community (Please replace the contents in parentheses by (i.e. not included by Guglielmone et al. 2010) only further contributes to confusion about the known range of a species or their involvement in the transmission of pathogens. Ideally, ticks should be collected and reported with specific reference to a pair of coordinates and time of collection(s), together with the pertinent information regarding stage(s) and host(s). It is ecologically meaningless to report the occurrence of ticks based solely on political divisions. Voucher specimens for every collection should be made available for future examination regarding the reliability in tick determination, especially when new tick-host relationships or novel pathogen connections are reported. In this regard, it is especially timely that studies on the distribution of ticks be undertaken to provide more information in view of drastic changes occurring in biocenoses over large areas due to increasing population pressures, social land reform measures and changing agricultural practices.

## Electronic supplementary material

Below is the link to the electronic supplementary material.

Supplementary material 1 (JPG 600 kb)

Supplementary material 2 (DOC 44 kb)

Supplementary material 3 (XLS 43 kb)

Supplementary material 4 (XLS 39 kb)
