# Supplementary figures and images for: Association of environmental traits with the geographic ranges of ticks (Acari: Ixodidae) of medical and veterinary importance in the western Palearctic. A digital data set
Source: Exp Appl Acarol. 2012 Jul 28;59(3):351–66. doi: 10.1007/s10493-012-9600-7 (PMC3557372; doi:10.1007/s10493-012-9600-7)

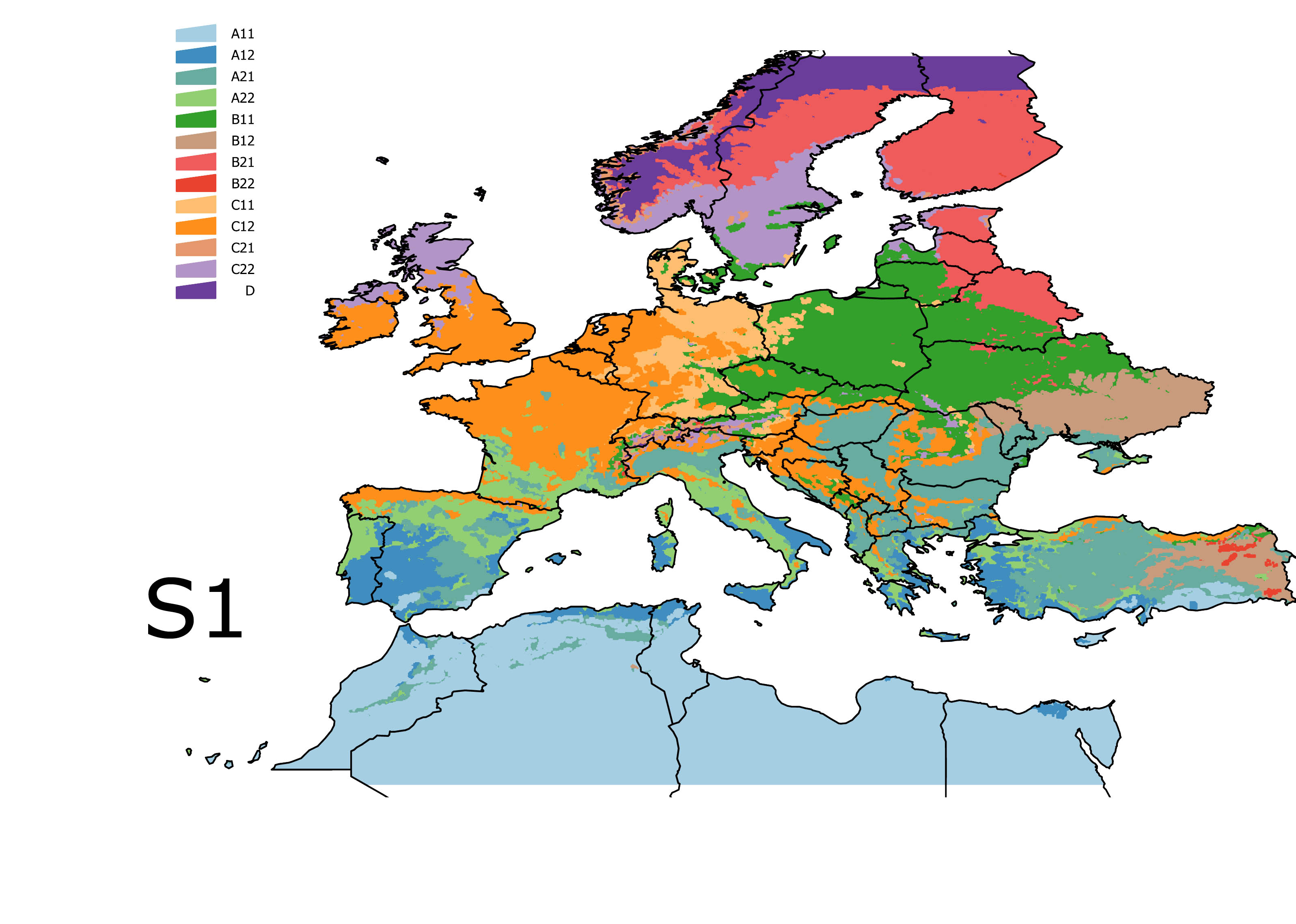

Supplement: Supplementary file 1 — Supplementary material 1 (JPG 600 kb) [file 10493_2012_9600_MOESM1_ESM.jpg]
