# Supplementary material for: Association of environmental traits with the geographic ranges of ticks (Acari: Ixodidae) of medical and veterinary importance in the western Palearctic. A digital data set
Source: Exp Appl Acarol. 2012 Jul 28;59(3):351–66. doi: 10.1007/s10493-012-9600-7 (PMC3557372; doi:10.1007/s10493-012-9600-7)
Supplement: Supplementary file 2 — Supplementary material 2 (DOC 44 kb) [file 10493_2012_9600_MOESM2_ESM.doc]

Table S1: Land Surface Temperature (T) and Normalized Derived Vegetation Index (NDVI) values for the categories of an unsupervised classification of the habitat, derived from remotely sensed, continuous monthly traits over the western Palearctic. Values of the mean ± standard error are included. Columns are minimum and maximum temperature (MinT, MaxT), minimum and maximum NDVI (MinNDVI, MaxNDVI) and the percentiles 20 and 80 (T20, T80) of both T and NDVI (NDVI20, NDVI80). Values for temperature are in degrees Kelvin (as available from MODIS imagery from which the data were extracted). NDVI values are unitless, between -1 and 1. The names to classify each signature are a consecutive labeling resulting from the hierarchical clustering algorithm.

| Category | MinT | MaxT | T20 | T80 | MinNDVI | MaxNDVI | NDVI20 | NDVI80 |
| --- | --- | --- | --- | --- | --- | --- | --- | --- |
| A11 | 283.55 ± 1.87 | 307.85 ± 2.67 | 288.41 ± 1.88 | 302.99 ± 2.38 | 0.13 ± 0.04 | 0.29 ± 0.16 | 0.16 ± 0.06 | 0.26 ± 0.13 |
| A12 | 280.96 ± 3.14 | 302.65 ± 2.86 | 285.3 ± 2.36 | 298.31 ± 2.13 | 0.25 ± 0.16 | 0.55 ± 0.14 | 0.31 ± 0.15 | 0.49 ± 0.14 |
| A21 | 272.44 ± 3.11 | 300.23 ± 3.02 | 278 ± 2.87 | 294.67 ± 2.81 | 0.16 ± 0.05 | 0.45 ± 0.12 | 0.22 ± 0.06 | 0.39 ± 0.11 |
| A22 | 268.9 ± 12.77 | 290.17 ± 16 | 273.16 ± 13.4 | 285.9 ± 15.36 | 0.16 ± 0.13 | 0.31 ± 0.16 | 0.19 ± 0.14 | 0.28 ± 0.15 |
| B11 | 263.96 ± 2.17 | 292.81 ± 1.43 | 269.73 ± 1.86 | 287.04 ± 1.36 | 0.15 ± 0.07 | 0.55 ± 0.1 | 0.23 ± 0.07 | 0.47 ± 0.09 |
| B12 | 263.1 ± 1.97 | 298.27 ± 2.76 | 270.13 ± 1.68 | 291.24 ± 2.25 | 0.1 ± 0.09 | 0.42 ± 0.13 | 0.16 ± 0.1 | 0.36 ± 0.12 |
| B21 | 255.87 ± 1.49 | 290.17 ± 2.53 | 262.73 ± 1.14 | 283.31 ± 1.95 | 0.16 ± 0.11 | 0.6 ± 0.06 | 0.25 ± 0.09 | 0.52 ± 0.06 |
| B22 | 259.71 ± 1.3 | 291 ± 2.6 | 265.97 ± 1.02 | 284.74 ± 2.02 | -0.04 ± 0.07 | 0.45 ± 0.09 | 0.06 ± 0.06 | 0.35 ± 0.07 |
| C11 | 256.52 ± 9.26 | 279.44 ± 10 | 261.11 ± 9.34 | 274.85 ± 9.84 | 0.13 ± 0.19 | 0.26 ± 0.32 | 0.16 ± 0.21 | 0.24 ± 0.29 |
| C12 | 270.4 ± 1.23 | 291.51 ± 1.89 | 274.62 ± 0.65 | 287.29 ± 1.28 | 0.26 ± 0.1 | 0.54 ± 0.17 | 0.32 ± 0.1 | 0.49 ± 0.15 |
| C21 | 260.19 ± 6.13 | 282.82 ± 5.1 | 264.72 ± 5.92 | 278.29 ± 5.3 | -0.02 ± 0.06 | 0.38 ± 0.19 | 0.06 ± 0.07 | 0.3 ± 0.16 |
| C22 | 263.49 ± 2.75 | 284.59 ± 2.38 | 267.71 ± 2.38 | 280.37 ± 2.13 | 0.2 ± 0.13 | 0.52 ± 0.1 | 0.27 ± 0.12 | 0.46 ± 0.11 |
| D | 250.83 ± 9.07 | 274.29 ± 11.9 | 255.52 ± 9.55 | 269.6 ± 11.27 | -0.06 ± 0.08 | 0.21 ± 0.23 | -0.01 ± 0.07 | 0.16 ± 0.18 |
